# Drivers of power line collisions and electrocutions of birds in Nepal

**DOI:** 10.1002/ece3.10080

**Published:** 2023-05-08

**Authors:** Suman Hamal, Hari Prasad Sharma, Ramji Gautam, Hem Bahadur Katuwal

**Affiliations:** ^1^ Central Department of Zoology, Institute of Science and Technology Tribhuvan University Kathmandu Nepal; ^2^ Nepal Zoological Society Kathmandu Nepal; ^3^ Prithvi Narayan Multiple Campus Tribhuvan University Pokhara Nepal; ^4^ Center for Integrative Conservation, Xishuangbanna Tropical Botanical Garden Chinese Academy of Sciences Mengla China

**Keywords:** agriculture lands, bird mortality, globally threatened birds, settlements, white‐rumped vulture

## Abstract

Among the several anthropogenic factors, power lines are increasingly regarded as one of the most significant hazards to bird species, primarily owing to collisions and electrocutions. Nepal has comparatively fewer studies on the impact of power line collisions and electrocution on birds compared with developed nations. From November 2021 to May 2022, we assessed the effect of power line collisions and electrocutions on the mortality of birds in the Putalibazar Municipality of the Syangja district of Nepal. We established 117 circular plots in diverse habitats, including agricultural lands, forests, settlements, and river basins, along a 30.6 km long distribution line. Within 18 plots, we detected 43 fatalities of 11 species (17 individuals of six species due to collision and 26 individuals of eight species due to electrocution). House Swift (*Apus nipalensis*) and Common Myna (*Acridotheres tristis*) were the primary victims of the collision, whereas House Crow (*Corvus splendens*) and Rock Pigeon (*Columba livia*) were frequently observed electrocuted. We also recorded the electrocution of the critically endangered White‐rumped Vulture (*Gyps bengalensis*). The total rate of bird power line collisions per kilometer was 0.55 birds, while the total electrocution rate per 10 poles was 2.22. The bird abundance, distance to agricultural regions, and proximity to human settlements were found to have a strong relationship with the mortality of birds caused by power lines. In order to reduce power line collisions and electrocution fatalities, we recommend conducting a detailed bird population study prior to determining the route of distribution lines.

## Graphical abstract

This study identified the bird collision and electrocution from distribution lines, which may be utilized for formulating policies and programs in order to ensure the safety and conservation of avifauna in Nepal. It has significant contributions to the board issue for avian species.

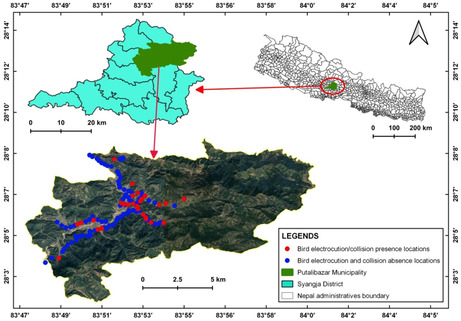

## 1 INTRODUCTION

Globally, the development of power lines is expanding significantly. Usually, two types of power lines are constructed, that is, transmission lines, the high‐tension wires that carry electricity with a magnitude larger than 60 kV from the power plant to substations, and distribution lines, the high‐tension wires that carry electricity with a magnitude between 1 and 60 kV (APLIC, 2012). Due to their distribution above the ground, birds are susceptible to collisions and electrocutions during flight and perching. Around 12–64 million birds perish annually in the United States due to conflict with power lines (Loss et al., 2014). Similarly, 2.5–25.6 million birds die annually in Canada due to direct collisions with power lines (Rioux et al., 2013). Power line collisions occur when birds fly into wires, whereas electrocution at poles occurs when a bird completes a circuit by touching two or more energized parts or an energized part and the grounded part (Real et al., 2001). Electrocution predominantly happens on distribution lines, but collisions can occur on both distribution and transmission lines (Dwyer et al., 2016; Lehman et al., 2007). The collision of power lines and electrocution represent a highly consequential anthropogenic hazard that is frequently overlooked, yet significantly contributes to bird mortality (Cornwall & Hochbaum, 1971; Guil & Pérez‐garcía, 2022; McNeil et al., 1985; Scott et al., 1972).

The issues of bird collision and electrocution are multifaceted, encompassing biological, topographical, meteorological, and technical aspects of power lines (APLIC, 2012). The susceptibility of certain bird species to hazards related to power lines may also be influenced by the characteristics of their surrounding habitats (Escobar‐Ibáñez et al., 2022). The agricultural landscapes that serve as important foraging and roosting areas for numerous bird species also pose heightened risks of collision and electrocution (Collins et al., 2010; Sundar & Choudhury, 2005). Conversely, it is difficult to locate casualties in forests, and collision and electrocution incidents are also relatively uncommon in forests, possibly due to the increased availability of natural perches (Benson, 1981; Switzer, 1977). Power lines traversing through human settlements carry an elevated risk of electrocution to bird populations, while power lines that traverse wetlands, marshes, rivers, lakes, and ponds are more likely to result in collisions (Gális et al., 2019). Lack of natural perching spots, such as tall trees, in the vicinity of power lines can force birds to rest on power poles or wires, ultimately increasing the likelihood of electrocution (Khadka, 2022). The incidence of power line collisions varies among the habitats, for example, 113 collisions/km/year in grasslands, 58 collisions/km/year in agricultural lands, and 489 collisions/km/year near river crossings were estimated in the Netherlands in the 1980s (Erickson et al., 2005). The fluctuation in the population and spatial distribution of bird species during breeding, migratory, or wintering periods has a significant impact on the incidence rates of electrocution and collision (APLIC, 2006).

Large‐ and medium‐sized diurnal birds, such as eagles, hawks, vultures, kites, and falcons, are the most common electrocution victims (Ferrer, 2012; Fransson et al., 2019; Gadzhiev, 2013; Guil et al., 2011; Haas, 2005; Prinsen et al., 2011; Škorpíková et al., 2020). Due to their broad wingspan, diurnal raptors (e.g., Black Kite *Milvus migrans*) and waterbirds (e.g., Cattle Egret *Bubulcus ibis*, Sarus Crane *Grus antigone*) are susceptible to electrocution (APLIC, 2006; Janss & Ferrer, 2001; Sundar & Choudhury, 2005). Agricultural and suburban areas have a higher incidence of corvids electrocution due to their increased population size, a larger body size propensity to use power poles for perching (APLIC, 2006). Whereas, passerines are less susceptible to electrocution due to their relatively smaller size but they are vulnerable to power line collisions due to their low aspect ratio (i.e., short wings and short tail) and swift maneuverability (Janss, 2000). Endangered species such as *Gyps* vultures are especially prone to fatal power line hazards due to their frequent use of power line towers as roost sites and vantage points (Mundy et al., 1992) with an estimated 4% decline in the local population of the Cape vultures (*G. coprotheres*) per year in the Eastern Cape Province of Southern Africa (Boshoff et al., 2011). Between 2010 and 2021, 15 bird species including vultures (144 individuals) have been electrocuted in Nepal as a result of the construction of electricity distribution lines without a proper assessment of their long‐term impact on the environment (Bhusal, 2022; Chaudhary et al., 2019; Rasaili, 2022). Rare and endangered species with delayed mortality and low reproductive rates may experience population decline even if fewer adults are killed by power lines (Janss, 2000; Mundy et al., 1992). Therefore, in order to prevent bird fatality, it is imperative that thorough research be conducted to identify and understand the multiple factors that influence collisions and electrocutions.

Underground cabling of the low and medium voltage power lines is quite common in most developed countries, including Belgium, Germany, the Netherlands, Norway, and the USA, which is a boon to the bird's population with no risks of collision and electrocution (Haas, 2005). Although the subterranean cabling is theoretically viable, the price of installing it can be 4–10 times greater than the building of regular overhead lines. As consequences, above‐ground power lines are abundantly constructed in most of the developing countries including Nepal. However, the specific data on the impacts of distribution lines on bird species in Nepal are rarely understood. Therefore, we aimed to provide the baseline data on bird collision and electrocution from distribution lines and understand the factors affecting power line collisions and electrocutions in putalibazar municipality. Our findings will add to formulating policies and programs in order to ensure the safety and conservation of avifauna in Nepal.

## 2 MATERIALS AND METHODS

### 2.1 Study area

**FIGURE 1:**
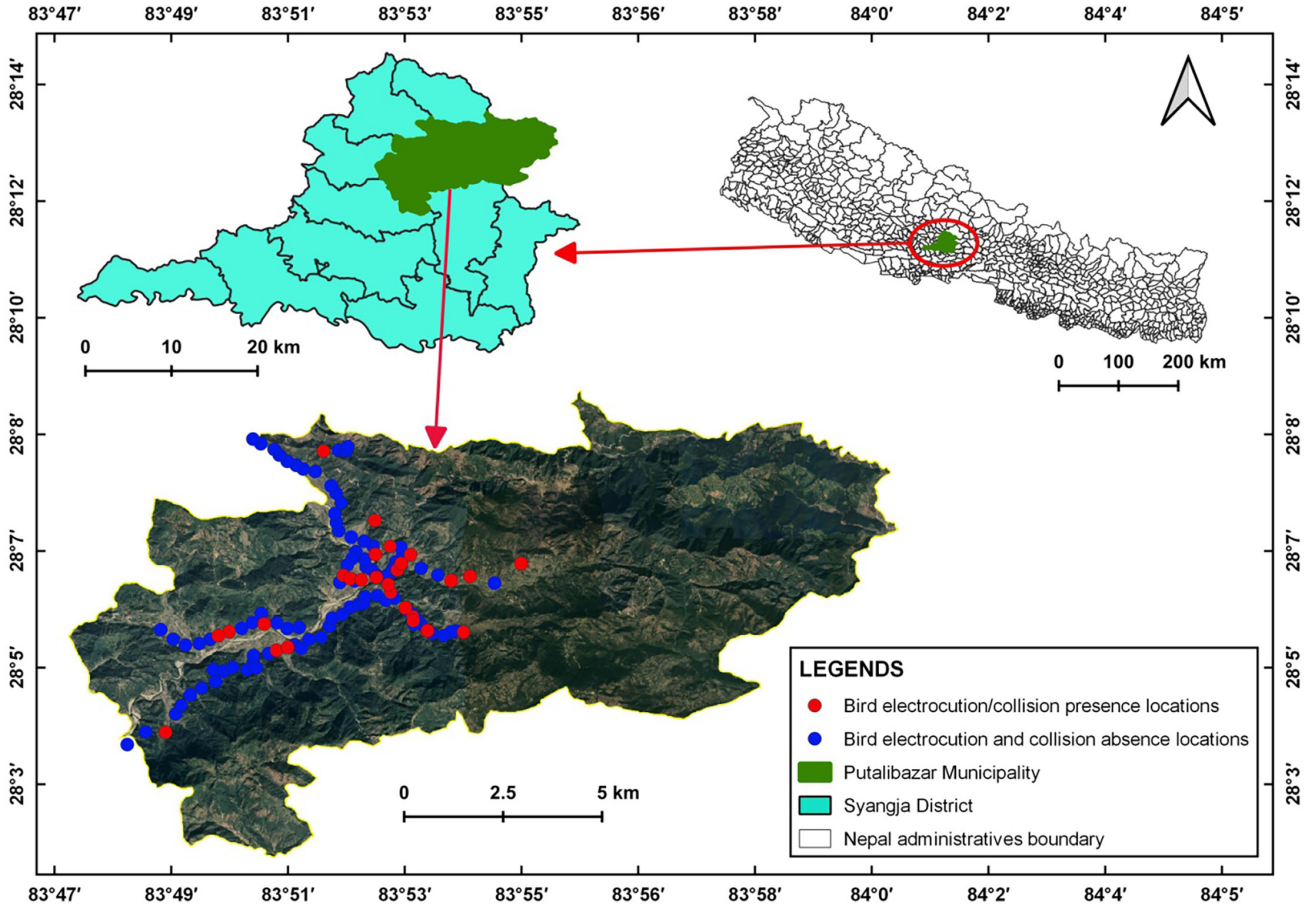
Avian Mortality location due to power lines collision and electrocution in Putalibazar Municipality from November 2021 to May 2022.

We conducted this study in Putalibazar Municipality, the district headquarter of Syangja district of Gandaki Province (Figure 1). It has an area of 147.21 km^2^ and extends from 28°03′18″N to 28°08′44″N and 83°47′30″E to 83°54′34″E, with a total human population of 41,743 individuals (CBS, 2023). The municipality is divided into 14 smaller administrative units called wards. The elevation of the municipality is 836 m from the sea level. This urban municipality came into existence in 1997 and was restructured on March 12, 2017 (GoN, 2017).

The distribution of birds in the study site is not uniform and varies on habitat type, geographic factors, and human effects. The primary vegetation types of the study area include Sal (*Shorea robusta*), Pine (*Pinus roxburgii*), Khair (*Senegalia catechu*), Dhale Katus (*Castanopsis indica*), and mixed forest (Bhandari et al., 2018). The primary crops grown in this region include paddy, maize, wheat, millet, pulses, and potatoes. Fruit trees include citrus, banana, guava, jackfruit, and peach (CBS, 2012). Although there is no detailed exploration of the wildlife in the municipality, the Kalij Pheasant (*Lophura leucomelanos*), Red Jungle Fowl (*Gallus gallus*), Cattle Egret (*Bubulcus ibis*), House Crow (*Corvus splendens*), Common Myna (*Acridotheres tristis*), etc. are regularly encountered birds.

### 2.2 Research design

Developmental activities are rapidly increasing in the Syangja district. More importantly, Kali Gandaki “A” and Andhikhola hydropower stations are located in the district. The distribution lines of these two hydropower stations pass through the Putalibazar municipality. We conducted this study along the distribution lines located in Putalibazar municipality that extends through the settlements, highways, agricultural lands, river basins, and forests (Figure 1).

We did a preliminary survey from October 10 to October 12, 2021 to determine the routes of distribution lines in Putalibazar municipality. District Electricity Authority, Syangja also provided us with information on the distribution line voltages, such as low (11 kV) with a single disc insulator per phase and high (33 kV) with three disc insulators per phase. In addition, both types of distribution lines had pin‐type insulators at power poles. Phase‐to‐phase clearances are approximately 28 cm for 11 kV and 64 cm for 33 kV (NEA, 2019). The pole's height for the 11 kV line ranges from 4.6 to 5.8 m and for 33 kV ranges from 5.2 to 6.1 m depending upon their location (DoED, 1993). For distribution lines of 11–33 kV, the distance between two consecutive power poles, also known as the span length, is approximately 50 m (NEA, 2022). For bird survey, we chose every 6th pole to establish the point count stations. So the distance between two point count stations was around 300 m, the generally used technique for bird survey (Bibby et al., 2000). Altogether, we used a total of seven segments of low voltage distribution lines and eight segments of high voltage distribution lines with an average length of 900 m (±648.07 m SD) and 3037.5 m (±1752.5 m SD), respectively, for bird survey.

We established a circular plot of 10 m radius at each sixth pole (point count station) to count the bird richness, abundance, nests and bird electrocution, and collision‐related data. Before bird observation, we spent 5 min in each plot to reduce the disturbances. We counted birds for 10 min in each plot. For electrocution incidents of birds, we recorded the bird carcasses within a radius of 10 m from the pole. We confirmed the death with or without burn marks on the feathers, feet, or bill as electrocution victims. For collision data, we walked slowly and carefully, observing the site within 10 m on both sides of the power line for any incident of injury or death of the birds. We confirmed the death of birds due to collision as noticed by injuries on the body such as broken bones, wings, legs and shoulder bones, and if the carcasses were found just below or within 10 m on both sides of phase conductors (power line) (Gális et al., 2019). All the identified bird carcasses were removed away from the area to avoid double counting. The research was conducted between 6:00 a.m. and 11:00 a.m. The birds were observed using the Cason 8 × 40 binocular and identified using Grimmett et al. (2016). We collected bird data from November 8, 2021 to May 12, 2022 and visited each station four times. We also further asked local people about collision and electrocution fatalities to account for the maximum incidents and to fill the gap in our study time. We collected data from 30.6‐km‐long distribution line at 117 sites. A low voltage distribution line, or 11 kV, encompassed 28 study locations. High voltage distribution line, that is, 33 kV covered 89 study sites. In addition, we also recorded number of trees (>2 m) and measured forest canopy from the center of each plot using a spherical densitometer for each plot. We also categorized the habitats in each plot, such as agricultural lands (*n* = 48), forests (*n* = 29), settlements (*n* = 25), and river basins (*n* = 15). For each plot, we also measured the nearest distance to the agricultural lands, forest, settlements, and water sources using a measuring tape (<200 m) and GIS (>200 m).

### 2.3 Data analysis

We used a Generalized Linear Model (GLM) with a binomial distribution to determine the variables influencing power line collisions and electrocutions. We categorized each plot as either present (record of bird collision and electrocution) or absent (no incidences of bird collision and electrocution) and considered it as the response variable. The nearest distance to agricultural land, forest, settlement, and water source, as well as voltage (low and high), bird abundance, forest canopy cover, and trees present, were used as predictor factors. Prior to undertaking GLM, we ran a correlation analysis among the predictor variables and omitted those that were highly associated with *r* > .70 in the same model (Libal et al., 2011). In correlation analysis, number of trees and forest canopy cover were substantially associated (ǀ*r*ǀ = .76). Therefore, number of trees was removed from the analysis. All analyses were performed in R software (R Core Team, 2019).

## 3 RESULTS

We recorded 19 species of 853 individuals of birds representing 14 families and nine orders along the distribution line during the study period (Table A1 in Appendix 1). The mean (±SD) nest count in the trees located within the observation plot was 0.06 ± 0.329 [range: 0–2]. The mean (±SD) distance from the observation plot to the nearest agricultural land was 35.09 ± 48.38 m [range: 0–258 m], forest was 46.18 ± 54.72 m [range: 0–292 m], settlement was 51.66 ± 60.75 m [range: 0–292 m], and water source was 175.4 ± 172.21 m [range: 4–712 m].

### 3.1 Power line collision and electrocution mortality

Altogether, we recorded 11 species (*n* = 43) of birds for collision‐ and electrocution‐related mortality (Table 1). The average carcass count for the observation plot was 0.367 ± 0.772 (SD) [range: 0–4]. We recorded 17 individuals of six species due to a power line collision (Table 1). The collision rates of Common Myna (*Acridotheres tristis*; *n* = 6) and House Swift (*Apus nipalensis*; *n* = 5) were the greatest (Table 1). The total rate of bird power line collisions was 0.55 birds/km. In case of electrocution, a total of 26 electrocuted birds of eight species were recorded, and they were found in 15% of plots (*n* = 18) (Table 1). The most electrocuted species was House Crow (*Corvus splendens*; *n* = 11) followed by Rock Pigeon (*Columba livia*; *n* = 5; Table 1). We also recorded the electrocution of the critically endangered White‐rumped Vulture (*Gyps bengalensis*). We noticed higher bird collision and electrocution incidences in high voltage lines (*n* = 34) than in low voltage lines (*n* = 9). Similarly, we found greater collision and electrocution rates in agricultural fields (*n* = 21), followed by settlement (*n* = 15), and the lowest rates in river basins (*n* = 5) and forests (*n* = 2).

**TABLE 1:** Birds mortality by electrocution and collision in distribution lines in Putalibazar Municipality from November 2021 to May 2022.

Species	Collision	Electrocution	Collision/km	Electrocution/10 poles	IUCN status
*Acridotheres tristis*	6	1	0.19	0.09	LC
*Apus nipalensis*	5	0	0.16	0.00	LC
*Bubulcus ibis*	0	4	0.00	0.34	LC
*Columba livia*	1	5	0.03	0.43	LC
*Corvus macrorhynchos*	0	1	0.00	0.09	LC
*Corvus splendens*	2	11	0.07	0.94	LC
*Ficedula superciliaris*	1	0	0.03	0.00	LC
*Gyps bengalensis*	0	1	0.00	0.08	CR
*Milvus migrans*	0	2	0.00	0.17	LC
*Myophonus caeruleus*	0	1	0.00	0.08	LC
*Passer domesticus*	2	0	0.07	0.00	LC
	17	26	0.55	2.22	

### 3.2 Factors affecting the power line collisions and electrocutions

**FIGURE 2:**
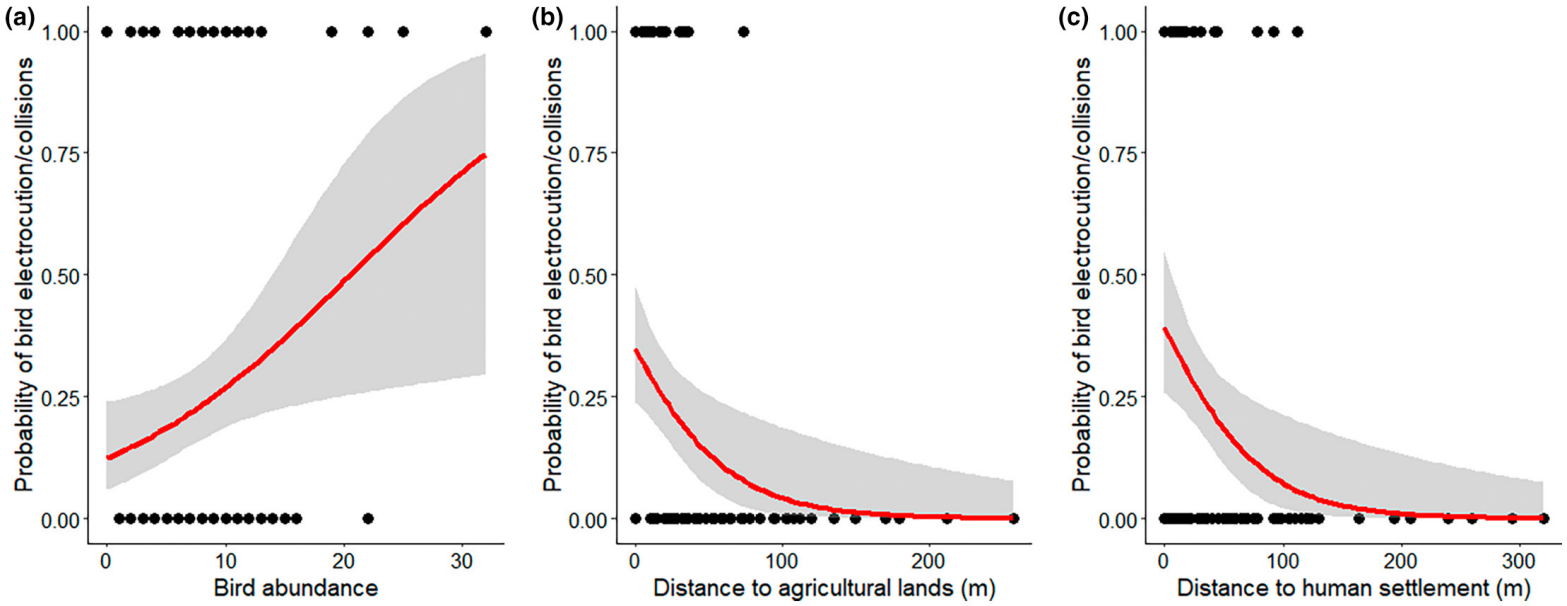
Response of bird abundance, proximity to agricultural land, and human settlement on bird electrocutions and power line collisions.

The probability of power line collisions and electrocutions was greatly impacted by bird abundance and the proximity to agricultural lands and human settlements (Table 2). The probability of an incident was higher in places with higher bird abundance and near agricultural lands and settlements (Table 2; Figure 2a–c).

**TABLE 2:** Generalized linear model describing the effects of given predictors on power line‐related bird mortality in Putalibazar Municipality from November 2021 to May 2022.

Parameters	Estimate	SE	*Z*	*p*
Intercept	−0.471	0.670	−0.703	.482
Voltage: low	−0.367	0.662	−0.554	.580
Forest canopy cover	0.003	0.011	0.297	.766
Bird abundance	0.128	0.053	2.412	**.016**
Distance to water source	0.000	0.002	−0.259	.796
Distance to agricultural land	−0.030	0.013	−2.278	**.023**
Distance to forest	−0.002	0.005	−0.298	.765
Distance to settlement	−0.030	0.011	−2.823	**.005**

*Note*: Model parameters include voltage (low or high), forest canopy cover (%), bird abundance, distance to water (m), distance to agricultural lands (m), distance to forest (m), and distance to settlement (m). Significant effects are in bold.

## 4 DISCUSSION

Our research indicates that collisions with power lines and electrocution have an adverse influence on some bird species in the Putalibazar Municipality. Although greater numbers were found of globally least concern species, mortality occurrences were also reported for globally threatened species, indicating that birds of Nepal are extremely sensitive to collisions with power lines and electrocution.

We discovered higher numbers of victims of Common Myna and House Swift in our study area, probably due to the large population of these species, as also reported in Spain (Janss, 2000). House crows and Rock Pigeons were the most electrocuted bird species, presumably due to their greater abundance and longer wingspan to complete the circuit between any two energized components (Janss, 2000). High voltage, namely 33 kV, was found to have a higher bird fatality than low voltage. However, our sampling was more extensive in the 33 kV line, covering a distance of 24.3 km and resulting in 34 bird deaths, whereas the 11 kV line, spanning 6.3 km, recorded nine bird deaths. Although large birds such as raptors, storks, and owls are more prone to short‐circuit when resting if they come into touch with many conducting wires (Battaglini & Bätjer, 2015), our study shows that small‐sized birds such as flycatchers or swifts are also affected by power lines.

The most important factor determining bird collision and electrocution is the abundance of the birds (Bevanger, 1994). The higher the bird population, the higher the possibility of mortality, which might be attributed to the high density and behavioral characteristics of species (Lehman et al., 2007). Therefore, while constructing the route for the distribution lines, it is necessary to monitor the bird abundance and composition prior to avoid undesired collision and electrocution. As agricultural lands and human settlements provide important habitats for various bird species (Grimmett et al., 2016; Katuwal et al., 2022), we observed an increase in collision and electrocution fatalities in the vicinity of agricultural land and human settlements. This might be due to the fact that birds utilize the power lines and poles within these ecosystems as perching sites while they forage for prey (Perez‐Garcia et al., 2011). In addition to that, large number of power lines is built on agricultural land due to suitable conditions for the installation of utility structures (Dixon et al., 2017; Siriwardena et al., 1998; Wretenberg et al., 2006). Power lines in close proximity to bird concentration habitats, such as wetlands and agricultural land, are the most hazardous because birds establish breeding and wintering colonies and concentrate at higher densities, which dramatically increases the likelihood of collision (Andriushchenko & Popenko, 2012; Faanes, 1987; Malcolm, 1982). For example, around 1% of Sarus Crane (*Grus antigone*) population dies annually due electricity wires‐related incidents in India (Sundar & Choudhury, 2005). Collision and electrocution‐related mortality are also documented in the forest. However, it is difficult to discover the carcass, particularly in the forest and also in river basins, due to the abundance of scavengers and the fact that they are more difficult to detect than on agricultural land or in human settlements. Therefore, careful route planning is one of the most effective ways to reduce bird collisions with overhead power lines (D'Amico et al., 2018). We recommend attachment of markers onto the power lines in the form of plates, spirals, flappers, swivels, or spheres to increase their visibility are by far the most common mitigation measure applied to reduce bird collisions with power lines (APLIC, 2012; Barrientos et al., 2011; Prinsen et al., 2012). While the use of avian safe pole designed with sufficient separation between energized phase conductors (also called “phases”) and between phases and grounded hardware to accommodate at least the wrist‐to‐wrist or head‐to‐foot distance of a bird, insulation of exposed part or wire and installation of perch management techniques will help to reduce the electrocution incidents (Prinsen et al., 2012).

Our study has some limitations because it was conducted in a short period of time, which could have an effect on the rate of power line collision and electrocution incidents. We conducted bird surveys within a 10 m radius and not at all times of the year, which reduced the richness and abundance of bird species. We only visited the region four times between November and May, thus we may have missed some incidents, as the carcasses may have been taken or consumed by predators. Despite the fact that we consulted with the locals, we believe that it is insufficient. Hence, the number of power line collision and electrocution incidences may be greater in our study area than we reported. As a result, we advise continuous monitoring or monitoring at least once a week in order to identify all possible incidents.

## 5 CONCLUSION

We conclude that power line collisions and electrocutions are imposing substantial threats to the common and the threatened birds in Putalibazar Municipality. The chance of bird mortality owing to power lines was found to be significantly related to bird abundance and the distance to agricultural lands, and human habitation. We advocate for careful route planning and timely supervision of distribution lines, as well as the deployment of appropriate measures in order to decrease the likely risks of collision and electrocution to birds.

## AUTHOR CONTRIBUTIONS

**Suman Hamal:** Conceptualization (equal); data curation (equal); formal analysis (equal); investigation (equal); methodology (equal); writing – original draft (equal); writing – review and editing (equal). **Hari Prasad Sharma:** Conceptualization (equal); data curation (equal); formal analysis (equal); methodology (equal); project administration (equal); supervision (equal); writing – original draft (equal); writing – review and editing (equal). **Ramji Gautam:** Conceptualization (equal); writing – review and editing (equal). **Hem Bahadur Katuwal:** Writing – review and editing (equal).

## CONFLICT OF INTEREST STATEMENT

The authors declare no conflict of interest.

## Data Availability

The data generated during this study are deposited to Dryad 10.5061/dryad.6m905qg3s.

## APPENDIX 1

**TABLE A1:** Total birds recorded in the plots during the study period at Putalibazar Municipality.

S. No.	Common name	Scientific name	Order	Family	IUCN	National Red List
1	Black Bulbul	*Hypsipetes leucocephalus*	Passeriformes	Pycnonotidae	LC	LC
2	Black Drongo	*Dicrurus macrocercus*	Passeriformes	Dicruridae	LC	LC
3	Black Kite	*Milvus migrans*	Accipitriformes	Accipitridae	LC	LC
4	Blue Whistling‐thrush	*Myophonus caeruleus*	Passeriformes	Muscicapidae	LC	LC
5	Cattle Egret	*Bubulcus ibis*	Pelecaniformes	Ardeidae	LC	LC
6	Common King Fisher	*Alcedo atthis*	Coraciformes	Alcedinidae	LC	LC
7	Common Myna	*Acridotheres tristis*	Passeriformes	Sturnidae	LC	LC
8	House Crow	*Corvus splendens*	Passeriformes	Corvidae	LC	LC
9	House Sparrow	*Passer domesticus*	Passeriformes	Passeridae	LC	LC
10	House Swift	*Apus nipalensis*	Caprimulgiformes	Apodidae	LC	LC
11	Large‐billed Crow	*Corvus macrorhynchos*	Passseriformes	Corvidae	LC	LC
12	Little Egret	*Egretta garzetta*	Pelecaniformes	Ardeidae	LC	LC
13	Red Jungle fowl	*Gallus gallus*	Galliformes	Phasianidae	LC	LC
14	Rock Pigeon	*Columba livia*	Columbiformes	Columbidae	LC	LC
15	Rose‐ringed Parakeet	*Alexandrinus krameri*	Psittaciformes	Psittaculidae	LC	LC
16	Ultramarine Flycatcher	*Ficedula superciliaris*	Passeriformes	Muscicapidae	LC	LC
17	Western Koel	*Eudynamys scolopaceus*	Cuculiformes	Cuculidae	LC	LC
18	Western Spotted Dove	*Spilopelia suratensis*	Columbiformes	Columbidae	LC	LC
19	White‐rumped Vulture	*Gyps bengalensis*	Accipitriformes	Accipitridae	CR	CR

Abbreviations: CR, critically endangered; IUCN, International Union for Conservation of Nature; LC, least concern.
